# Comprehensive analysis of a TNF family based-signature in diffuse gliomas with regard to prognosis and immune significance

**DOI:** 10.1186/s12964-021-00814-y

**Published:** 2022-01-09

**Authors:** Qiang-Wei Wang, Wei-Wei Lin, Yong-Jian Zhu

**Affiliations:** 1 Department of Neurosurgery, The Second Affiliated Hospital, Zhejiang University School of Medicine, No. 88 Jiefang Road, Hangzhou, 310009 China; 2 Chinese Glioma Genome Atlas Network (CGGA) and Asian Glioma Genome Atlas Network (AGGA), Beijing, 100070 China

**Keywords:** Glioma, Tumor necrosis factor, Prognosis, Tumor immunity

## Abstract

**Background:**

Several studies have shown that members of the tumor necrosis factor (TNF) family play an important role in cancer immunoregulation, and trials targeting these molecules are already underway. Our study aimed to integrate and analyze the expression patterns and clinical significance of TNF family-related genes in gliomas.

**Methods:**

A total of 1749 gliomas from 4 datasets were enrolled in our study, including the Cancer Genome Atlas (TCGA) dataset as the training cohort and the other three datasets (CGGA, GSE16011, and Rembrandt) as validation cohorts. Clinical information, RNA expression data, and genomic profile were collected for analysis. We screened the signature gene set by Cox proportional hazards modelling. We evaluated the prognostic value of the signature by Kaplan–Meier analysis and timeROC curve. Gene Ontology (GO) and Gene set enrichment analysis (GSEA) analysis were performed for functional annotation. CIBERSORT algorithm and inflammatory metagenes were used to reveal immune characteristics.

**Results:**

In gliomas, the expression of most TNF family members was positively correlated. Univariate analysis showed that most TNF family members were related to the overall survival of patients. Then through the LASSO regression model, we developed a TNF family-based signature, which was related to clinical, molecular, and genetic characteristics of patients with glioma. Moreover, the signature was found to be an independent prognostic marker through survival curve analysis and Cox regression analysis. Furthermore, a nomogram prognostic model was constructed to predict individual survival rates at 1, 3 and 5 years. Functional annotation analysis revealed that the immune and inflammatory response pathways were enriched in the high-risk group. Immunological analysis showed the immunosuppressive status in the high-risk group.

**Conclusions:**

We developed a TNF family-based signature to predict the prognosis of patients with glioma.

Video abstract

**Supplementary Information:**

The online version contains supplementary material available at 10.1186/s12964-021-00814-y.

## Background

Gliomas are the most common primary malignant tumors in the adult central nervous system [1, 2]. Despite accounting for less than 2% of newly diagnosed tumors, gliomas are associated with high malignancy and mortality [3, 4]. Glioblastoma, WHO grade 4 glioma, is one of the most challenging malignancies. The current standard treatment for glioblastoma is total surgical resection combined with radiotherapy and chemotherapy, which only extends the median survival of patients to 14.6 months [5, 6]. In order to improve the prognosis of patients, it is urgent to further understand the pathogenesis of gliomas and develop novel treatment strategies, such as targeted therapy and immunotherapy. Immune checkpoint blockade therapy enhances the anti-tumor immune response by targeting the regulatory pathways in immune cells. In recent years, checkpoint blockade of the CTLA-4 or PD-1/PD-L1 axis has been proven to be an effective strategy for advanced melanoma, non-small-cell lung carcinoma, advanced renal cell carcinoma and Hodgkin lymphoma [7–9].

Tumor necrosis factor (TNF) family consists of 19 ligands of TNF superfamily (TNFSF) and 29 members of TNF receptors superfamily (TNFRSF), which affect many biological processes, including apoptosis, host defense, inflammation, and autoimmunity [10]. TNF is produced by many different immune and non-immune cell types, which plays an important role in the development and function of the immune system [11]. At present, in addition to blocking the immune checkpoints of the B7-CD28 family (such as PD1/PD-L1), anti-tumor immunity can also be augmented by biologics or genetic engineering techniques that modulate TNFSF/TNFRSF signaling [12, 13]. Some cancer immunotherapy targets from the TNF family are very attractive and have entered the stage of clinical trials, such as 4-1BB, OX40, GITR, and so on [14–16].

In gliomas, some members of the TNF family have been widely studied for their role in regulating tumor genesis and growth. Yeung et al. revealed that TNF-α promoted the production of multiple inflammatory mediators by p38 MAPK signaling, thereby contributing to the expansion of GBM [17]. Ramaswamy et al. found that TNF-α enhanced the invasion ability of glioma cells through MEK-ERK signaling [18]. Shibahara et al. demonstrated that the OX40/OX40L (TNFRSF4/ TNFSF4) signaling pathway induced anti-tumor immunity in a mouse glioma model, and OX40 could also trigger regulatory T cells to cause immunosuppression under hypoxia [19]. The members of TNF family might activate or inhibit immune responses in the tumor microenvironment [13], so some members have been selected as potential targets for glioma immunotherapy. Woroniecka et al. revealed that 4-1BB (TNFRSF9) agonism reduced exhaustion of tumor-infiltrating lymphocytes and improved their function, thereby prolonging the survival of GBM in combination with anti-PD1 therapy [20]. Shoji et al. found that the local delivery of an anti-CD40 (TNFRSF5) agonistic antibody induced significant anti-tumor effects in mouse glioma models [21]. The agonist OX40 (TNFRSF4) immunotherapy combined with vaccination reversed T lymphocyte exhaustion and prolonged survival in the glioma mouse model [22]. The TNF family has shown great potential in targeted therapy. However, currently, the characteristic of TNF family-related gene set has not been systematically profiled in gliomas.

In this study, we systematically analyzed the expression patterns and clinical significance of TNF family-related genes in gliomas. We developed a TNF family-based risk signature that classified patients into the high-risk or low-risk group. There were significant differences in clinicopathological characteristics, genomic alteration, prognosis, and immune status between the two groups. Finally, we established an individualized nomogram model based on signature to predict the 1-year, 3-year, and 5-year overall survival rate of glioma patients.

## Methods

### Data collection

Our study collected 1749 glioma cases from four public datasets. As the training set, the Cancer Genome Atlas (TCGA, http://cancergenome.nih.gov/) dataset contained RNA-seq data, somatic mutation, copy-number alterations (CNAs), clinical and pathological information of 702 glioma cases. The validation sets contained our Chinese Glioma Genome Atlas (CGGA) dataset, GSE16011 dataset, and Rembrandt dataset. In our CGGA dataset, we have collected RNA-seq data of 325 gliomas, which were generated by the Illumina HiSeq platform [23]. We also collected clinical and molecular information of CGGA patients. Our CGGA dataset was approved by the Beijing Tiantan Hospital Capital Medical University Institutional Review Board (IRB KY2013-017-01) [24]. The other two validation sets included 268 glioma cases from the GSE16011 microarray database (http://www.ncbi.nlm.nih.gov/geo/query/acc.cgi?acc=GSE16011) and 454 glioma cases from The Repository for Molecular Brain Neoplasia Data (Rembrandt, https://www.ncbi.nlm.nih.gov/geo/query/acc.cgi?acc=GSE108474).

### Signature development

We included all well-defined TNF family genes, including 18 TNFSF genes and 29 TNFRSF genes. In the training set, univariate Cox proportional hazards regression analysis was used to screen TNF family genes related to overall survival. With selected genes, the Least Absolute Shrinkage and Selection Operator (LASSO) regression algorithm generated a Cox model with minimum average cross validation error based on tenfold cross validation [25–27]. The LASSO cox model included 8 genes and our signature risk score was developed with a linear combination of 8 gene expression level (expr) weighted by their LASSO regression coefficients: Risk Score = (expr_gene1_ × coefficient_gene1_) + (expr_gene2_ × coefficient_gene2_) + ⋯ + (expr_gene8_ × coefficient_gene8_).

### DAVID functional annotation and Gene Set Enrichment Analysis (GSEA)

DAVID (https://david.ncifcrf.gov/) is a comprehensive set of functional annotation tool for understanding the biological meaning behind gene sets. We first performed Pearson correlation analysis and screened out genes that were significantly positively correlated with signature (Pearson R > 0.6, p < 0.05). Then these genes were uploaded to DAVID for Gene Ontology (GO) and Kyoto Encyclopedia of Genes and Genomes (KEGG) pathway enrichment analysis.

Gene set enrichment analysis (GSEA) is a widely used tool for assessing pathway enrichment with transcriptome data. In this study, we adopted FGSEA (Fast Gene Set Enrichment Analysis) method, which could estimate low GSEA P-values with a high accuracy in a short time [28]. |NES| > 1 and adjusted p-value < 0.05 were considered significant in GSEA.

### Analysis of immune and inflammatory responses

The CIBERSORT algorithm allowed us to quantify the infiltrating immune cells in tumors with gene expression profiles [29]. We calculated 22 immune cell subtypes with the customized gene signature file “LM22”. Differences in the proportion of immune cells between high-risk and low-risk groups were assessed by Student’s t-test.

We analyzed seven inflammatory metagenes, including 104 genes [30]. Metagenes were calculated by Gene Sets Variation Analysis (GSVA) [31] using the corresponding gene sets.

### Statistical analysis

R language (v4.0.0, https://www.r-project.org/) was the main statistical analysis environment. Normalized gene expression values were log-transformed (based on 2). Univariate and multivariate Cox regression analysis were performed to evaluate the prognostic value. Differences in variables between the groups were assessed using Student’s t-test or Chi-square test. The survival differences in Kaplan–Meier survival curves were evaluated by log-rank test. Time-dependent ROC curve (timeROC) was used to predict one-, three- and five-year overall survival [32, 33]. The nomogram model integrated signature and clinical indicators to predict 1-year, 3-year and 5-year survival probability with R package “rms” [34]. Other R packages involved in this study included pheatmap, gglpot2, pROC, ComplexHeatmap, Hmisc, circlize and corrgram. A two-sided test p value < 0.05 was considered statistically significant. P values were adjusted by Benjamini–Hochberg procedure (BH) in multiple hypothesis testing.

## Results

### The landscape and prognostic value of the TNF family in gliomas

Totally, 47 well-defined TNF family genes were enrolled in our study, including 18 TNFSF genes and 29 TNFRSF genes. First, we analyzed the gene expression of the TNF family in 702 glioma patients from TCGA dataset. Pearson correlation analysis of TNF family genes showed significant positive correlations among most genes (Additional file 1: Fig. S1). Then, univariate Cox regression analysis was used to evaluate the association between TNF family genes and overall survival of glioma patients. Among all TNF family genes, 39 genes were found to be significantly related to overall survival, including 25 TNFRSF genes and 14 TNFSF genes (adjusted p < 0.05, Table 1). We found that four genes (*EDA*, *TNFRSF21*, *TNFRSF13C*, *EDAR*) were protective, with hazard ratios (HR) less than 1. And 35 genes (*TNFRSF12A*, *TNFRSF11B*, *TNFSF14*, *TNFRSF14*, *TNFRSF1A*, *LTBR*, *CD70*, *CD40*, *TNFRSF19*, *FAS*, *TNFRSF10C*, *TNFRSF4*, *NGFR*, *TNFRSF10B*, *TNFSF4*, *TNFRSF18*, *CD40LG*, *TNFRSF10D*, *TNFSF11*, *FASLG*, *TNFSF13*, *TNFSF8*, *TNFSF10*, *TNFSF13B*, *TNFSF12*, *TNFRSF6B*, *TNFRSF9*, *TNFRSF1B*, *LTB*, *TNFSF15*, *TNFRSF11A*, *TNFRSF10A*, *CD27*, *EDA2R*, *RELT*) were risk factors, with HR greater than 1.

**Table 1:** Univariate Cox analysis of TNF family genes in TCGA dataset

Official symbol	Aliases	Family	HR	95%CI	waldtest-P	Adjusted P
TNFRSF12A	FN14, TWEAKR, CD266	TNFRSF	1.7301	1.5910–1.8814	1.32E−37	6.204E−36
TNFRSF11B	OPG	TNFRSF	1.5674	1.4619–1.6805	1.24E−36	2.914E−35
TNFSF14	LIGHT, HVEML, CD258	TNFSF	1.6579	1.5221–1.8057	4.06E−31	6.36067E−30
TNFRSF14	LIGHTR, HVEM, CD270	TNFRSF	2.2535	1.9584–2.5932	7.94E−30	9.3295E−29
TNFRSF1A	TNFR1, CD120A	TNFRSF	2.0662	1.8156–2.3515	3.86E−28	3.6284E−27
LTBR	TNFRSF3	TNFRSF	2.1937	1.8974–2.5363	2.66E−26	2.08367E−25
CD70	TNFSF7, CD27L	TNFSF	1.3428	1.2696–1.4201	5.98E−25	4.01514E−24
CD40	TNFRSF5	TNFRSF	2.0981	1.8041–2.4399	6.42E−22	3.77175E−21
TNFRSF19	TROY, TAJ	TNFRSF	1.8957	1.6623–2.1619	1.41E−21	7.36333E−21
FAS	TNFRSF6, CD95	TNFRSF	1.7655	1.5692–1.9863	3.35E−21	1.5745E−20
TNFRSF10C	TRAILR3, CD263	TNFRSF	1.8903	1.6539–2.1605	9.51E−21	4.06336E−20
TNFRSF4	OX40, CD134	TNFRSF	1.5291	1.3893–1.6830	3.95E−18	1.54708E−17
NGFR	TNFRSF16, CD271	TNFRSF	1.3981	1.2926–1.5122	5.84E−17	2.11138E−16
TNFRSF10B	TRAILR2, CD262	TNFRSF	2.0613	1.7288–2.4578	7.70E−16	2.585E−15
TNFSF4	OX-40L, CD134L, CD252	TNFSF	1.8707	1.6029–2.1832	1.93E−15	6.04733E−15
TNFRSF18	GITR, AITR, CD357	TNFRSF	1.5424	1.3852–1.7175	2.78E−15	8.16625E−15
CD40LG	TNFSF5, CD154	TNFSF	1.6220	1.4332–1.8357	1.85E−14	5.11471E−14
TNFRSF10D	TRAILR4, CD264	TNFRSF	1.5565	1.3895–1.7435	2.16E−14	5.64E−14
TNFSF11	RANKL, CD254	TNFSF	1.8101	1.5532–2.1095	3.02E−14	7.47053E−14
FASLG	TNFSF6, CD95-L	TNFSF	1.5788	1.3972–1.7839	2.36E−13	5.546E−13
TNFSF13	APRIL, CD256	TNFSF	1.9666	1.6316–2.3703	1.26E−12	2.82E−12
TNFSF8	CD30L, CD153	TNFSF	1.5178	1.3472–1.7101	6.97E−12	1.48905E−11
TNFSF10	TRAIL, CD253	TNFSF	1.5351	1.3504–1.7451	5.63E−11	1.15048E−10
TNFSF13B	BAFF, CD257	TNFSF	1.3137	1.2087–1.4279	1.37E−10	2.68292E−10
EDA	EDA-A1, EDA-A2	TNFSF	0.6320	0.5450–0.7330	1.30E−09	2.444E−09
TNFRSF21	DR6, CD358	TNFRSF	0.6007	0.5083–0.7099	2.22E−09	4.01308E−09
TNFRSF13C	BAFFR, CD268	TNFRSF	0.6751	0.5934–0.7681	2.38E−09	4.14296E−09
TNFSF12	TWEAK	TNFSF	2.1779	1.6714–2.8377	8.21E−09	1.37811E−08
TNFRSF6B	DCR3	TNFRSF	1.4530	1.2672–1.6661	8.76E−08	1.41972E−07
TNFRSF9	4-1BB, CD137, ILA	TNFRSF	1.4266	1.2523–1.6252	9.14E−08	1.43193E−07
TNFRSF1B	TNFR2, CD120B	TNFRSF	1.4901	1.2851–1.7278	1.28E−07	1.94065E−07
LTB	TNFSF3	TNFSF	1.3777	1.2227–1.5524	1.43E−07	2.10031E−07
TNFSF15	TL1A	TNFSF	1.6125	1.3464–1.9312	2.07E−07	2.94818E−07
TNFRSF11A	RANK, CD265	TNFRSF	1.3585	1.2008–1.5370	1.14E−06	1.57588E−06
TNFRSF10A	TRAILR1, CD261	TNFRSF	1.4137	1.2197–1.6384	4.26E−06	5.72057E−06
CD27	TNFRSF7	TNFRSF	1.4331	1.2254–1.6761	6.67E−06	8.70806E−06
EDA2R	TNFRSF27, XEDAR	TNFRSF	1.1858	1.0997–1.2786	9.38E−06	1.19151E−05
EDAR	EDA-A1R	TNFRSF	0.7789	0.6889–0.8807	6.66E−05	8.23737E−05
RELT	TNFRSF19L	TNFRSF	1.6259	1.2176–2.1712	0.000987074	0.001189551
TNFRSF25	DR3, TNFRSF12	TNFRSF	1.1035	0.9905–1.2294	0.07383002	0.086750274
TNFSF18	GITRL	TNFSF	0.9237	0.8426–1.0127	0.090776101	0.104060408
TNFRSF13B	TACI, TNFRSF14B, CD267	TNFRSF	0.7812	0.5602–1.0893	0.145467208	0.162784733
LTA	TNFSF1	TNFSF	1.1115	0.9614–1.2851	0.153148522	0.167394896
TNFRSF8	CD30	TNFRSF	1.0540	0.9681–1.1475	0.225337476	0.240701395
TNF	TNFSF2, TNFA	TNFSF	0.9766	0.9050–1.0539	0.542331079	0.566434683
TNFRSF17	BCMA, TNFRSF13A, CD269	TNFRSF	1.0573	0.8585–1.3021	0.599972185	0.613015059
TNFSF9	4-1BB-L, CD137L	TNFSF	1.0003	0.8851–1.1304	0.996518328	0.996518328

### Identification of a TNF family based-signature in gliomas

**Fig. 1:**
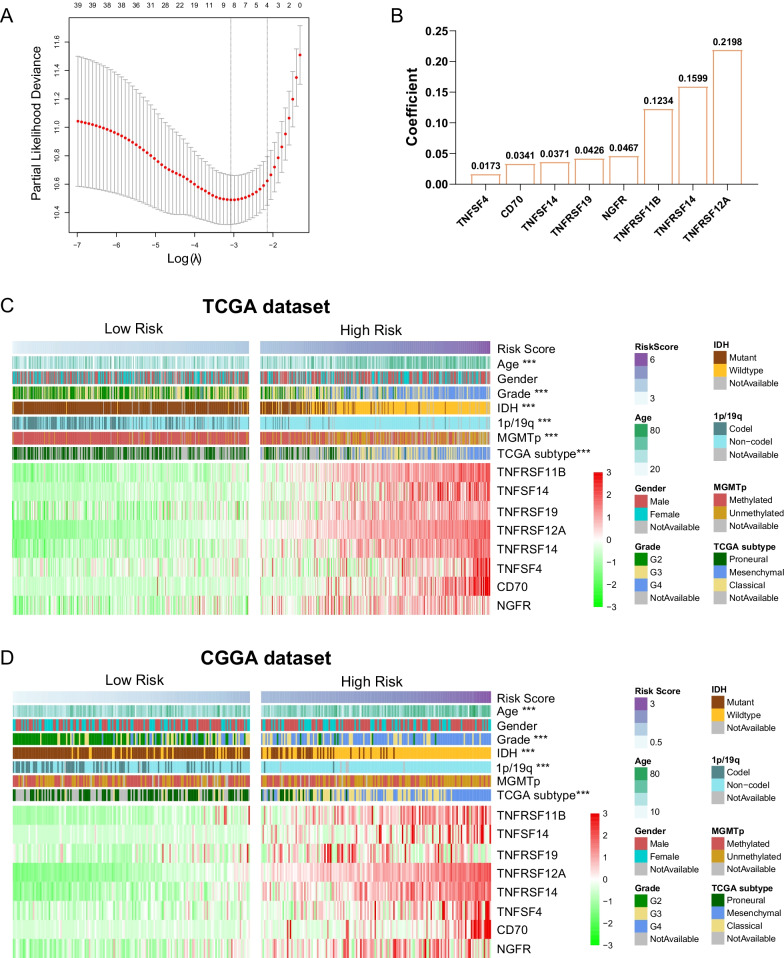
Identification of a TNF family-based signature in gliomas. **a** Cross validation for optimizing parameter screening by LASSO regression algorithm in the TCGA training dataset. **b** The regression coefficients of 8 genes screened by LASSO model. The heatmaps show the clinical and molecular features of the high-risk and low-risk groups in TCGA dataset (**c**) and CGGA dataset (**d**). P values are adjusted. **P < 0.01; ***P < 0.001. Abbreviations: IDH, isocitrate dehydrogenase; MGMT, methylguanine methyltransferase

After univariate Cox regression analysis, 39 genes were left for further analyzed in TCGA dataset. By LASSO regression algorithm, we screened eight genes as covariates to evaluate prognostic value (Fig. 1a). Our TNF family based-signature (risk score) was developed with a linear combination of the expression of eight genes weighted by their regression coefficients (Fig. 1b). Then, TCGA patients were divided into low-risk group and high-risk group according to the median risk score as the cutoff value. We found significant differences in clinical and pathological characteristics between the two groups (Fig. 1c and Table 2). Patients in the high-risk group were older than those in the low-risk group (p < 0.001). And the incidence of GBMs (WHO grade 4) in the high-risk group was higher than that in the low-risk group (p < 0.001). Meanwhile, IDH-wildtype, 1p/19q non-codeletion, and MGMT promoter unmethylation were more common in the high-risk group (p < 0.001). Malignant molecular subtypes, including classical and mesenchymal subtypes, were significantly enriched in the high-risk group (p < 0.001). In the validation dataset (CGGA, GSE16011 and Rembrandt datasets), the same regression coefficients from TCGA (Fig. 1b) were used to calculate the risk score of each patient. In three validation datasets, we also observed differences in clinical and pathological features between high-risk and low-risk group (Fig. 1d, Additional file 1: Figure S2, Table 2 and Additional file 1: Table S1).

**Table 2:** Correlation between RS and clinicopathological factors of glioma patients

Characteristics	TCGA dataset (n = 702)			CGGA dataset (n = 325)		
	Low-risk group (n = 351)	High-risk group (n = 351)	Adjusted p	Low-risk group (n = 162)	High-risk group (n = 163)	Adjusted p
Age						
Mean(range)	41 (14–74)	53 (21–89)	< 0.001	40 (10–75)	47 (8–81)	< 0.001
Gender						
Female	137	118	n.s	63	59	n.s
Male	163	191		99	104	
NA	51	42		0	0	
Grade						
2	174	42	< 0.001	96	9	< 0.001
3	124	117		36	40	
4	2	150		30	114	
NA	51	42		0	0	
IDH status						
Mutant	331	97	< 0.001	138	38	< 0.001
Wildtype	5	229		24	125	
NA	15	25		0	0	
1p/19q status						
Codel	155	14	< 0.001	65	2	< 0.001
Non-codel	182	313		94	156	
NA	14	24		3	5	
MGMT promoter						
Methylated	311	166	< 0.001	86	72	0.068
Unmethylated	27	135		64	85	
NA	13	50		12	6	
TCGA subtype						
Proneural	184	54	< 0.001	83	19	< 0.001
Classical	0	86		11	63	
Mesenchymal	0	95		0	68	
NA	167	116		68	13	

### Correlation between TNF family based-signature and pathological features in gliomas

**Fig. 2:**
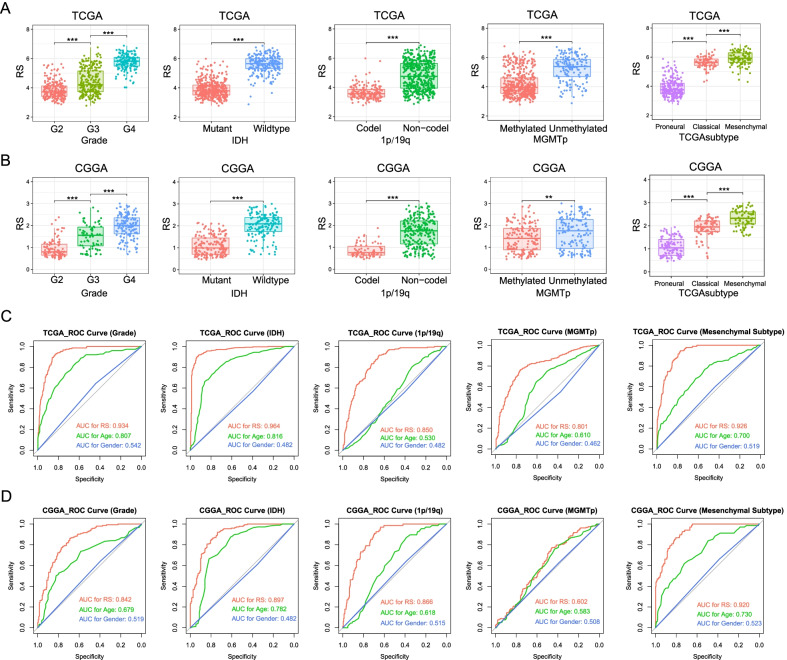
Association between pathological features and TNF family-based signature. The distribution of risk score (RS) in patients stratified by different pathological features (WHO Grade, IDH mutation and 1p/19q codeletion, MGMT promoter methylation, TCGA subtype) in TCGA dataset (**a**) and CGGA dataset (**b**). Receiver operating characteristic (ROC) curves show the predictive value of risk score, age and gender for pathological features (WHO Grade, IDH mutation and 1p/19q codeletion, MGMT promoter methylation, TCGA subtype) in TCGA dataset (**c**) and CGGA dataset (**d**). P values are adjusted. *P < 0.05; **P < 0.01; ***P < 0.001

Since gliomas covered grade 2–4 and different molecular subtypes, we further studied the distribution characteristics of TNF family based-signature (Fig. 2a). As the WHO grade increased from 2 to 4, risk score increased significantly (p < 0.05). Meanwhile, risk scores were significantly higher in the IDH-wildtype group, 1p/19q non-codeletion group, and the MGMT promoter unmethylated group (p < 0.05). Among the three TCGA subtypes, we found that patients defined as mesenchymal subtype had the highest risk score (p < 0.05). In all three validation datasets (Fig. 2b and Additional file 1: Fig. S3A and S3B), we also observed that the distribution of signature was consistent with the above results. Then, we utilized Receiver Operating Characteristic (ROC) curve to evaluate the predictive value of our signature for pathological indicators. Compared with age and gender, our signature showed superior predictive value in WHO grade (AUC = 0.934), IDH mutation status (AUC = 0.964), 1p/19q codeletion status (AUC = 0.850), MGMT promoter methylation status (AUC = 0.801) and mesenchymal subtype (AUC = 0.926) (Fig. 2c). Signature also showed high predictive power in all three validation datasets (Fig. 2d, Additional file 1: Fig. S3C and S3D).

### Different patterns of genomic alterations between low- and high-risk gliomas

**Fig. 3:**
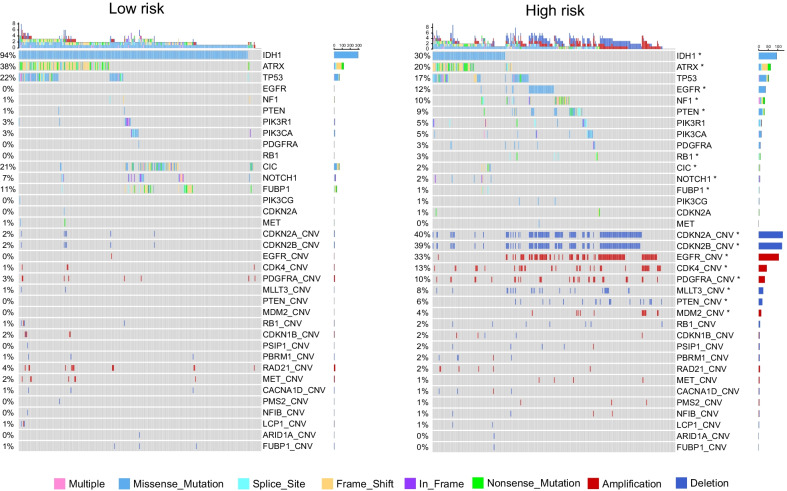
Analysis of genomic variation difference between low-risk and high-risk groups. The upper part of the oncoplots shows the somatic mutations analysis, and the lower part shows copy number alterations analysis. Chi-square test, p values are adjusted. *P < 0.05

At the level of genomic alterations, somatic mutation and copy-number alterations (CNA) data from TCGA were included for further study. In Fig. 3, we found a significant enrichment of *IDH1, ATRX, CIC, NOTCH1, and FUBP1* mutations in the low-risk group (p < 0.05). And mutations in *EGFR, NF1, PTEN,* and *RB1* were significantly enriched in the high-risk group (p < 0.05). CNA analysis showed that the high-risk group had more amplification regions such as *EGFR, CDK4, PDGFRA, MDM2*, and more deletion regions such as *CDKN2A, CDKN2B, MLLT3, PTEN* (Fig. 3).

### Prognostic analysis of the TNF family based-signature

**Fig. 4:**
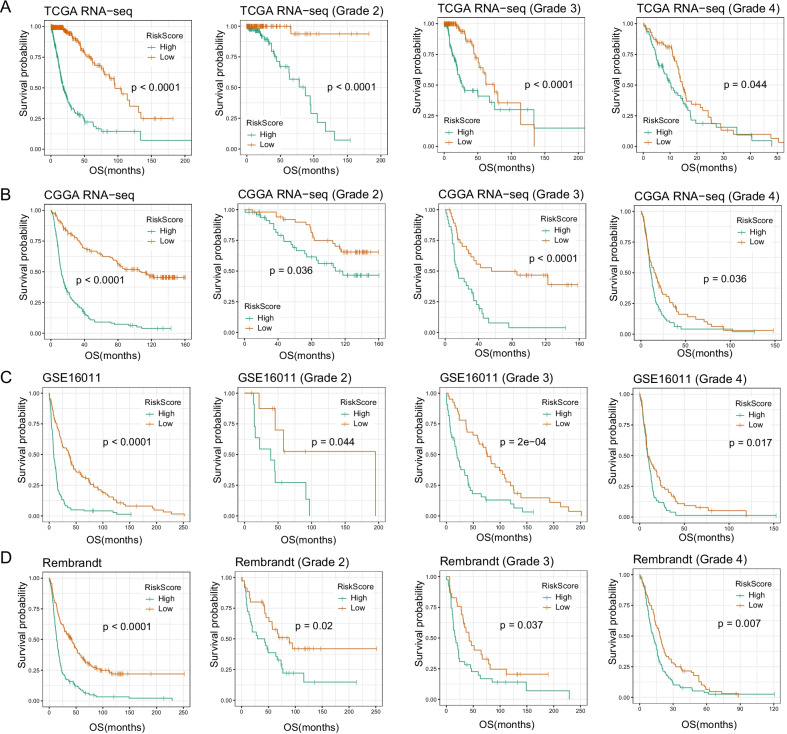
Survival analysis of the TNF family-based signature in gliomas. The survival curves show the difference in overall survival between the high-risk and low-risk groups in all grade, grade 2, grade 3, and grade 4 gliomas. **a** TCGA dataset, **b** CGGA dataset, **c** GSE16011 dataset, **d** Rembrandt dataset. P values are obtained by the log-rank test and are adjusted

Next, we evaluated the clinical prognostic value of our signature using follow-up data. The Kaplan–Meier survival curve showed that the high-risk group had a significantly worse prognosis than the low-risk group in four datasets (Fig. 4, p < 0.001, log-rank test). When patients were divided into WHO grade 2, grade 3 and grade 4, we also observed significantly shorter survival in the high-risk group than in the low-risk group (p < 0.05). In both univariate and multivariate Cox regression analyses, our signature risk score was significantly associated with overall survival in four datasets (Table 3, p < 0.05). This suggested our signature with independent prognostic value, independent of other clinicopathological factors (age, gender, WHO grade, IDH status, 1p/19q status, and MGMT promoter status).

**Table 3:** Variables related to OS in gliomas: univariate and multivariate analysis

	Univariate Cox regression			Multivariate Cox regression		
	HR	95% CI	Adjusted p	HR	95% CI	Adjusted p
*TCGA*						
Age Increasing years	1.075	1.063–1.088	< 2e−16*	1.062	1.045–1.079	2.376e−12*
Gender (male vs. female)	1.001	0.743–1.347	0.997			
Grade (GBM vs. LGG)	9.576	6.835–13.420	< 2e−16*	1.327	0.823–2.141	0.369
IDH (wild vs. mutant type)	11.070	7.772–15.770	< 2e−16*	1.212	0.612–2.402	0.631
1p/19q (non-codel vs. codel)	4.541	2.671–7.719	2.66e−08*	1.556	0.825–2.936	0.344
MGMT promoter (unmethylated vs. methylated)	3.207	2.312–4.447	4.032e−12*	1.100	0.745–1.623	0.631
RiskScore Increasing scores	3.516	2.945–4.197	< 2e−16*	2.317	1.670–3.213	1.449e−06*
*CGGA*						
Age Increasing years	1.035	1.023–1.048	3.794e−08*	1.015	1.003–1.027	0.016*
Gender (male vs. female)	0.998	0.759–1.312	0.988			
Grade (GBM vs. LGG)	4.919	3.670–6.593	< 2e−16*	2.286	1.629–3.209	4.3e−06*
IDH (wild vs. mutant type)	2.866	2.171–3.782	2.45e−13*	0.626	0.416–0.943	0.025*
1p/19q (non-codel vs. codel)	5.877	3.602–9.588	2.3275e−12*	3.027	1.778–5.156	7.58e−05*
MGMT promoter (unmethylated vs. methylated)	1.195	0.911–1.566	0.232			
RiskScore Increasing scores	3.360	2.737–4.126	< 2e−16*	2.319	1.692–3.178	8.4e−07*
*GSE16011*						
Age Increasing years	1.041	1.030–1.051	8.38e−14*	1.041	1.023–1.059	1.755e−05*
Gender (male vs. female)	1.066	0.811–1.401	0.647			
Grade (GBM vs. LGG)	3.131	2.353–4.166	1.476e−14*	1.360	0.767–2.409	0.292
IDH (wild vs. mutant type)	1.930	1.423–2.618	2.808e−05*	1.432	0.859–2.386	0.28
1p/19q (non-codel vs. codel)	2.445	1.645–3.633	1.452e−05*	1.354	0.813–2.255	0.292
RiskScore Increasing scores	3.956	2.875–5.442	< 2e−16*	3.110	1.393–6.946	0.015*
*Rembrandt*						
Gender (male vs. female)	1.105	0.828–1.475	0.496			
Grade (GBM vs. LGG)	2.671	2.060–3.464	2.48e−13*	1.815	1.077–3.057	0.038*
1p/19q (non-codel vs. codel)	2.348	1.287–4.285	0.007*	1.901	0.897–4.032	0.094
RiskScore Increasing scores	3.797	2.782–5.180	< 2e−16*	2.806	1.500–5.249	0.003*

HR, hazard ratio; CI, confidence interval; *Significant

### A survival prediction model based on the risk signature

**Fig. 5:**
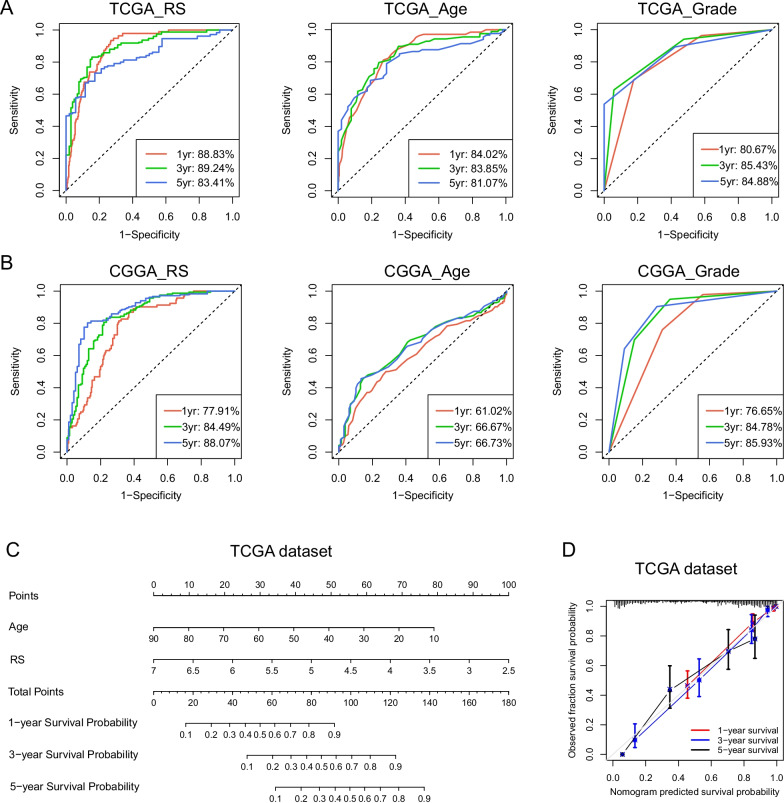
Development of an individualized survival prediction model based on TNF family-based signature. **a**, **b** The timeROC curves are used to evaluate the predictive ability of risk score, age and grade on 1-year, 3-year, and 5-year survival rates. **c** A nomogram model integrating the signature risk score and age in TCGA dataset. **d** Calibration curves of nomogram model for predicting 1-year (red line), 3-year (blue line) and 5-year (black line) overall survival

The ROC curve was performed to further evaluate the survival predictive value of signature. The 1-year, 3-year and 5-year AUC of signature were 88.83%, 89.24%, and 83.41%, superior to age (84.02%, 83.85%, 81.07%) and grade (80.67%, 85.43%, 84.88%) (Fig. 5a). In the three validation datasets, our signature also showed high time-dependent AUC (Fig. 5b and Additional file 1: Fig. S4). Combining independent prognostic indicators (age and risk score) in the TCGA dataset, we then constructed a nomogram model to predict 1-year, 3-year and 5-year survival probability for glioma patients (Fig. 5c, C-index = 0.868). The calibration diagram showed satisfactory consistency between the nomogram model prediction and observations in survival (Fig. 5d).

### Functional annotation of TNF family based-signature

**Fig. 6:**
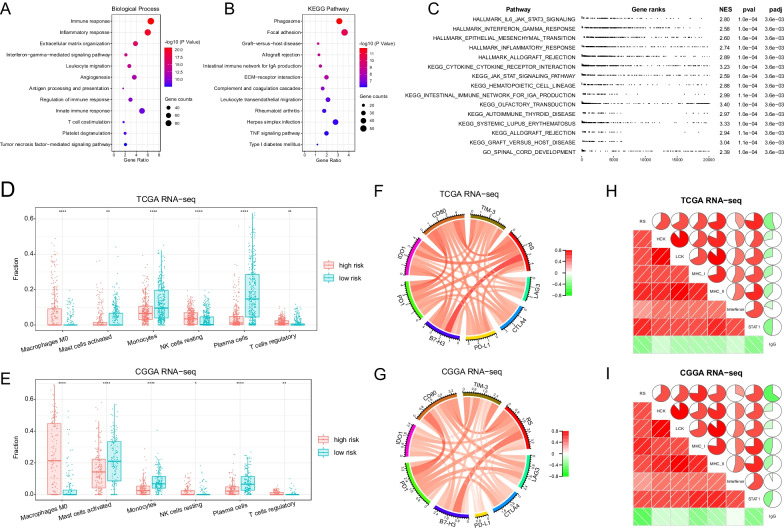
Functional annotation and immune landscape of TNF family-based signature in gliomas. Using GO terms of biological processes (**a**) and KEGG pathways (**b**), genes positively correlated with signature are functionally annotated. **c** Gene set enrichment analysis (GSEA) shows the top 15 pathways enriched in the high-risk group. **d**, **e** The CIBERSORT analysis shows different expression immune cells in the high-risk and low-risk groups. **f**, **g** Chord diagrams show correlation between risk score (RS) and eight immune checkpoint genes. Links show relations between objects (RS and eight immune checkpoint genes), and the width and color depth of links correspond to Pearson correlation coefficients. Red indicates a positive correlation and green indicates a negative correlation. **h**, **i** Corrgram plots show correlation between risk score and seven metagenes. In the upper pie chart and the lower shaded chart, red indicates a positive correlation and green indicates a negative correlation. The size of the pie chart and the intensity of the color are directly proportional to the correlation coefficient. P values are adjusted. *P < 0.05; **P < 0.01; ***P < 0.001; ****P < 0.0001

To reveal the underlying biological mechanism of TNF family based-signature, we screened 1578 genes that were significantly positively correlated with the signature (R > 0.6) through Pearson correlation analysis. Then we annotated the function of these genes in the DAVID online tool, and found that the most relevant biological processes included “immune response”, “inflammatory response”, “extracellular matrix organization”, “interferon-gamma-mediated signaling pathway”, and “leukocyte migration” (Fig. 6a). KEGG pathway analysis showed that these genes were enriched in immune-related pathways (Fig. 6b). Meanwhile, GSEA analysis showed enrichment of immune and inflammatory response pathways in the high-risk group, including “HALLMARK_IL6_JAK_STAT3_signaling” (NES = 2.80, padj = 3.6e−03), “HALLMARK_interferon_gamma_response” (NES = 2.58, padj = 3.6e−03), “HALLMARK_inflammatory_response” (NES = 2.74, padj = 3.6e−03), and “KEGG_cytokine_cytokine_receptor_interaction” (NES = 3.23, padj = 3.6e−03) (Fig. 6c).

### Association between the TNF family based-signature and glioma immune and inflammatory response

To better understand the relationship between TNF family and glioma immunity, we included immune cells and immune checkpoints for analysis. By CIBERSORT algorithm, we calculated 22 immune cell components in glioma samples. We found that in the high-risk group, suppressive or resting immune cells were significantly increased, including macrophages M0, resting NK cells, and regulatory T cells (Fig. 6d, e, p < 0.05). And the low-risk group had more activated immune cells, including activated mast cells, monocytes, and plasma cells. Meanwhile, we observed a significant correlation between risk score (RS) and proportion of six immune cell types (Figure S5, Pearson correlation, p < 0.05). In addition, eight common immune checkpoint genes (*PD-L1, PD1, LAG3, CTLA4, B7-H3, IDO1, CD80, TIM-3*) were selected for Pearson correlation analysis. The chord diagrams showed that signature risk score was positively correlated with the expression of all immune checkpoint genes in both TCGA and CGGA datasets (Fig. 6f, g), suggesting immunosuppression in the high-risk group.

Furthermore, we analyzed seven clusters of metagenes which represented different inflammatory responses. The corrgrams showed that the risk score was positively related to HCK, LCK, MHC_I, MHC_II, interferon, and STAT1, but was negatively related to IgG (Fig. 6h, i). These results suggested enrichment of antigen-presenting cells, macrophages, and T lymphocytes-related inflammatory responses, but not B lymphocytes, in the high-risk group.

## Discussion

As a new milestone in cancer treatment, modern immunotherapy has brought light to many tumors that previously had limited treatment options. Immunity checkpoint blockade with monoclonal antibodies targeting the B7-CD28 superfamily (CTLA-4, PD-1, and PD-L1) produces a durable anti-tumor immune response, and this strategy has been applied in many tumors and translated into clinical benefits [35]. However, some patients are resistant to immune checkpoint blockade therapy, and some patients will eventually relapse [36]. In lung cancer, the response to immune checkpoint blockade therapy is not universal, with more than half of patients showing primary resistance [37]. Among advanced melanoma that respond to CTLA-4 or PD-1 blockade therapy, approximately one-quarter to one-third of patients will relapse over time [38]. Also in gliomas, even though clinical trials of B7-CD28 superfamily inhibitors are now under active investigation, they have ended in failure so far and most patients have received little or no obvious benefit (CheckMate-143, CheckMate-498, CheckMate-548) [39–41]. These results suggest that there may be other immunoregulatory signaling pathways in the tumor microenvironment.

In fact, there are many pathways to protect tumor cells from immune damage, which is the recognition of self by immune cells, leading to resistance to the first generation of immune checkpoint blockers [42]. Therefore, it is necessary to develop new immunostimulatory targets to overcome the primary and acquired resistance of immunotherapy. In addition to the B7-CD28 family, the TNF family also contains many immune checkpoints, such as OX40, 4-1BB, GITR, and so on. In the process of antigen recognition, OX40 (TNFRSF4) is induced to express on activated T cells. Agonists targeting OX40 can provide powerful co-stimulatory signals, thereby enhancing the expansion and proliferation of CD4 + and CD8 + T cells that recognize tumor antigens. Several agonistic antibodies targeting OX40 are currently undergoing cancer clinical trials [15] and combining OX40 antibody and other immune checkpoint antibodies is more effective than monotherapy [43]. 4-1BB (TNFRSF9) is another attractive cancer immunotherapeutic target in the TNF family, which is a co-stimulatory receptor expressed on activated T cells and NK cells. The preclinical results of tumor models indicate that the currently developed agonist antibodies targeting 4-1BB can clear tumors and maintain durable anti-tumor immunity [44, 45]. Agonistic monoclonal antibodies targeting 4-1BB have shown good results in patients with lymphoma, and are undergoing combined therapy trials with other immunomodulators [14]. Due to the inflammatory hepatotoxicity of the first-generation 4-1BB antibody (urelumab), new 4-1BB agonists are under clinical development, seeking to maximize immune activation and avoid liver inflammation side effects [46].

In gliomas, immune checkpoints from the TNF family also serve as a co-stimulatory signal in regulating immunity. Woroniecka et al. found in mouse models that 4-1BB agonists eliminated the limitations of poor T cell activation and severe exhaustion, and combined anti-4-1BB and anti-PD1 treatments provided survival benefits [20]. Nusrat Jahan et al. found that the agonist anti-OX40 was effective for intracranial glioma and prolonged survival time in a mouse model of glioma [22]. In this study, we comprehensively analyzed the expression characteristics and prognostic significance of TNF family in gliomas. Pearson correlation analysis showed that the RNA expression of most TNF family genes was positively correlated. At the same time, univariate Cox regression analysis showed that the expression of 39 TNF family genes was significantly associated with overall survival. These results indicated that the TNF family members were closely related and had potential clinical value. Then using Lasso regression model, we developed a TNF family-based signature, which consisted of eight TNF family genes: *TNFSF4, CD70, TNFSF14, TNFRSF19, NGFR, TNFRSF11B, TNFRSF14,* and *TNFRSF12A*. Based on the median risk score, we divided patients into high-risk groups and low-risk groups, and assessed the differences between the two groups. We found that patients with older age, WHO grade 4, IDH-wildtype, 1p/19q intact, MGMT promoter non-methylated, and mesenchymal subtype were more common in the high-risk group. Meanwhile, we found a significant correlation between risk score (RS) and clinical molecular features (Figure S6, Spearman correlation, p < 0.05). At the level of genomic variation, we found that mutations in *EGFR, NF1, PTEN,* and *RB1* were significantly enriched in the high-risk group. And in the high-risk group, we found more amplification regions such as *EGFR, CDK4, PDGFRA, MDM2*, and deletion regions such as *CDKN2A, CDKN2B, MLLT3, PTEN*. These differences suggest that TNF family-based signature may be associated with the malignant biological process, as well as poor prognosis in glioma patients.

Next, survival curve analysis confirmed that patients in the high-risk group had a worse prognosis than low-risk group. And univariate and multivariate Cox regression analyses identified our signature as an independent prognostic indicator after adjustment of other clinicopathological factors. Then we used ROC curve to evaluate the survival predictive value of signature, which was superior to the traditional indicators (age and grade). Based on the superior predictive ability of signature, we combined with age to construct a nomogram survival prediction model. This model had good clinical application value in predicting the 1-year, 3-year and 5-year survival rates of individuals with gliomas.

In order to explore the potential biological mechanism of our signature, we performed DAVID functional annotation and GSEA enrichment analysis. We found differences in immune and inflammatory responses between the high-risk and low-risk groups, revealing the correlation between the TNF family and the immune microenvironment of gliomas. Then the immune cell infiltration analysis showed that the high-risk group had more suppressive or resting immune cells in, including macrophages M0, resting NK cells, and regulatory T cells. Meanwhile, signature was also positively correlated with the expression of immune checkpoints (*PD-L1, PD1, LAG3, CTLA4, B7-H3, IDO1, CD80, TIM-3*). These results all suggested the immunosuppressive status of the high-risk group. From these we could infer the close connection between the TNF family and the tumor immune microenvironment. TNF family members coordinately drove co-stimulation or co-inhibition of the immune response [47]. TNF family members usually exhibited the pro-inflammatory properties that were partly due to the activation of NF-kB signaling [48]. In addition, TNF members could activate immunosuppressive cells (regulatory T cells and myeloid-derived suppressor cells) through TNF receptor 2 (TNFR2), thus supporting immune escape and promoting tumor cell proliferation [49]. Although TNF members was initially found to mediate anti-tumor effects, recent studies have shown that they also promoted tumor progression. Lei et al. found that TNF-α treatment promoted the proliferation of glioma cells [50]. Wei et al. found that TNF-α secreted by macrophages could activate endothelial cells and promoted GBM angiogenesis [51]. In addition to the dual effects of TNF members, some members had synergistic effects. For example, CD27 (TNFRSF7), HVEM (TNFRSF14), 4-1BB (TNFRSF9) and OX40 (TNFRSF4) all had co-stimulatory effects on T cells, and the regulation of these co-stimulators might prolong T cell response and control the survival of T cells [52]. CD27 and HVEM expressed on resting T cells functioned early after initial activation of T cells, while OX40 and 4-1BB signals on T cells were delayed relative to initial activation and showed preferential effects on CD4 and CD8 T cells. The TNF family had diverse and complex interactions with the immune system [53], so the global evaluation of the TNF family was of great significance. Through bioinformatics analysis, we have systematically explored the TNF family-related genes and their potential relationship with immunity, but our study is preliminary and requires further in-depth analysis and biological experiment verification.

## Conclusions

In conclusion, this is the first study in the expression profile and clinical prognostic significance of TNF family members in gliomas. We also identified a TNF family-based signature to stratify the risk of glioma patients. Our research contributes to the individualized prognostic management of glioma patients, and provides evidence for immunotherapy targeting TNF family members.

## Supplementary Information

**Additional file 1**. **Figure S1.** The landscape of TNF family members in gliomas. **Figure S2.** Heatmap and clinicopathological features of low-risk and high-risk group in GSE16011 and Rembrandt dataset. **Figure S3.** Associations between TNF family-based signature and pathological features in GSE16011 and Rembrandt dataset. **Figure S4.** Predictive value of signature, age and grade for overall survival in GSE16011 and Rembrandt dataset. **Figure S5.** Corrplots show correlation between risk score (RS) and six immune cell types. **Figure S6.** Corrplots show correlation between risk score (RS) and clinical molecular features. **Table S1.** Correlation between RS and clinicopathological factors of glioma patients.

## Data Availability

The authors confirm the data that has been used in this work is available on reasonable request.

## Abbreviations

TCGA: The Cancer Genome Atlas
CGGA: Chinese Glioma Genome Atlas
TNF: Tumor necrosis factor
LGG: Lower grade gliomas
GBM: Glioblastomas
CNAs: Copy-number alterations
LASSO: Least absolute shrinkage and selection operator
GO: Gene ontology
GSEA: Gene set enrichment analysis
IDH: Isocitrate dehydrogenases
MGMT: O6-methylguanine-DNA methyltransferase
