# Supplementary material for: Comprehensive analysis of a TNF family based-signature in diffuse gliomas with regard to prognosis and immune significance
Source: Cell Commun Signal. 2022 Jan 9;20:6. doi: 10.1186/s12964-021-00814-y (PMC8744324; doi:10.1186/s12964-021-00814-y)
Supplement: Supplementary file 2 — Additional file 1. Figure S1. The landscape of TNF family members in gliomas. Figure S2. Heatmap and clinicopathological features of low-risk and high-risk group in GSE16011 and Rembrandt dataset. Figure S3. Associations between TNF family-based signature and pathological features in GSE16011 and Rembrandt dataset. Figure S4. Predictive value of signature, age and grade for overall survival in GSE16011 and Rembrandt dataset. Figure S5. Corrplots show correlation between risk score (RS) and six immune cell types. Figure S6. Corrplots show correlation between risk score (RS) and clinical molecular features. Table S1. Correlation between RS and clinicopathological factors of glioma patients. [file 12964_2021_814_MOESM2_ESM.docx]

**Figure S1. The landscape of TNF family members in gliomas.** Cellular interactions of TNF family members in gliomas. The green cells are from TNFSF and the orange cells are from TNFRSF. The size of each cell represents the influence of each member on overall survival (Wald test p value). The line connecting the cells represents the correlation between the two members. Red indicates positive correlation and blue indicates negative correlation.

**
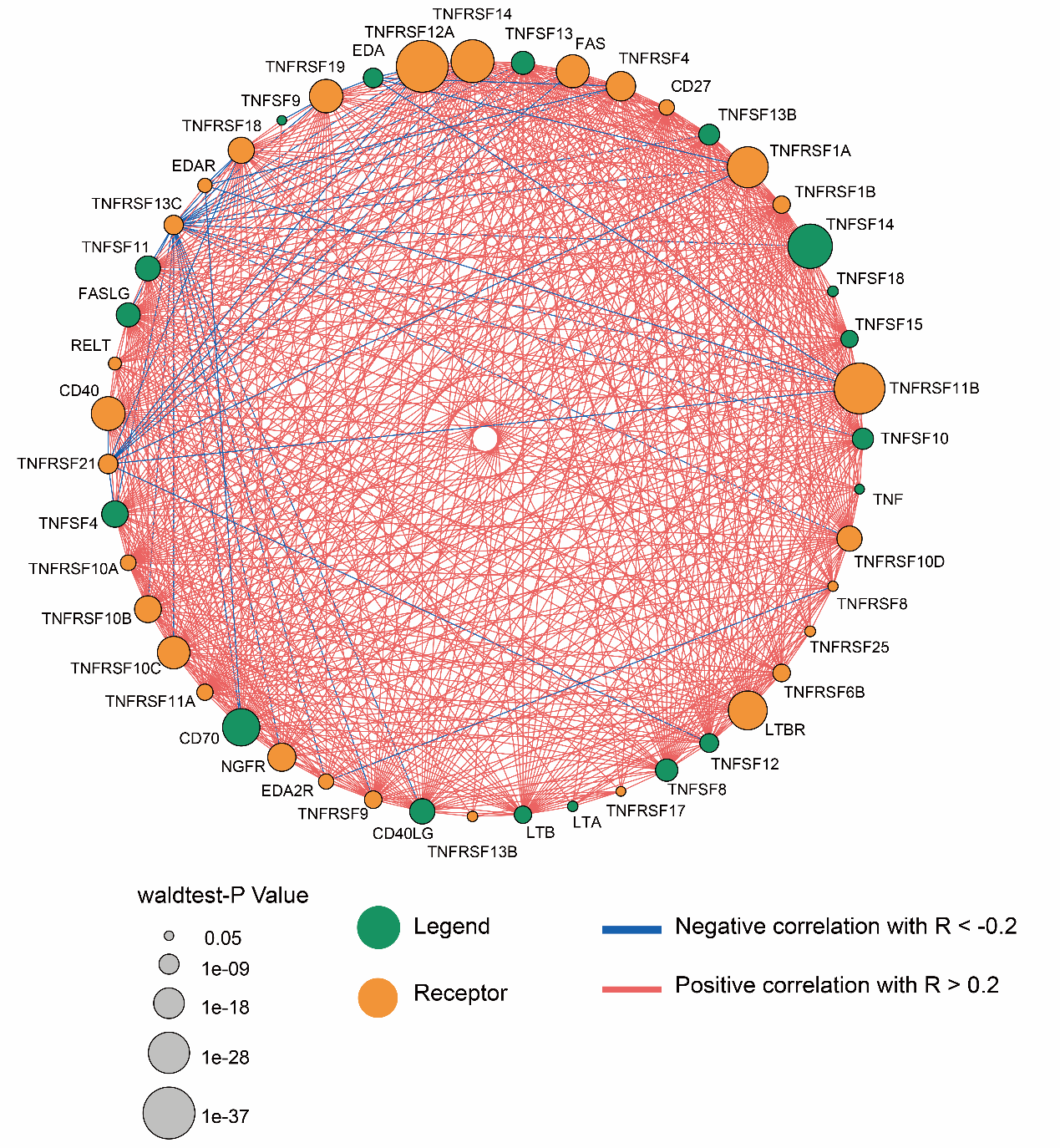
**

**Figure S2. Heatmap and clinicopathological features of low-risk and high-risk group in GSE16011 and Rembrandt dataset.** ***P < 0.001.

**Figure S3**. **Associations between TNF family-based signature and pathological features in GSE16011 and Rembrandt dataset.** (**A**) and (**B**) Patients were grouped by WHO grade, IDH mutation status, 1p/19q codeletion status, TCGA molecular subtype. (**C**) and (**D**) The ROC curve evaluated the predictive value of risk score in pathological features. *P <0.05, **P < 0.01, ***P < 0.001.

**Figure S4. Predictive value of signature, age and grade for overall survival in GSE16011 and Rembrandt dataset.** (**A**) 1-year, 3-year and 5-year ROC curves indicated the sensitivity and specificity of risk score, age and grade.


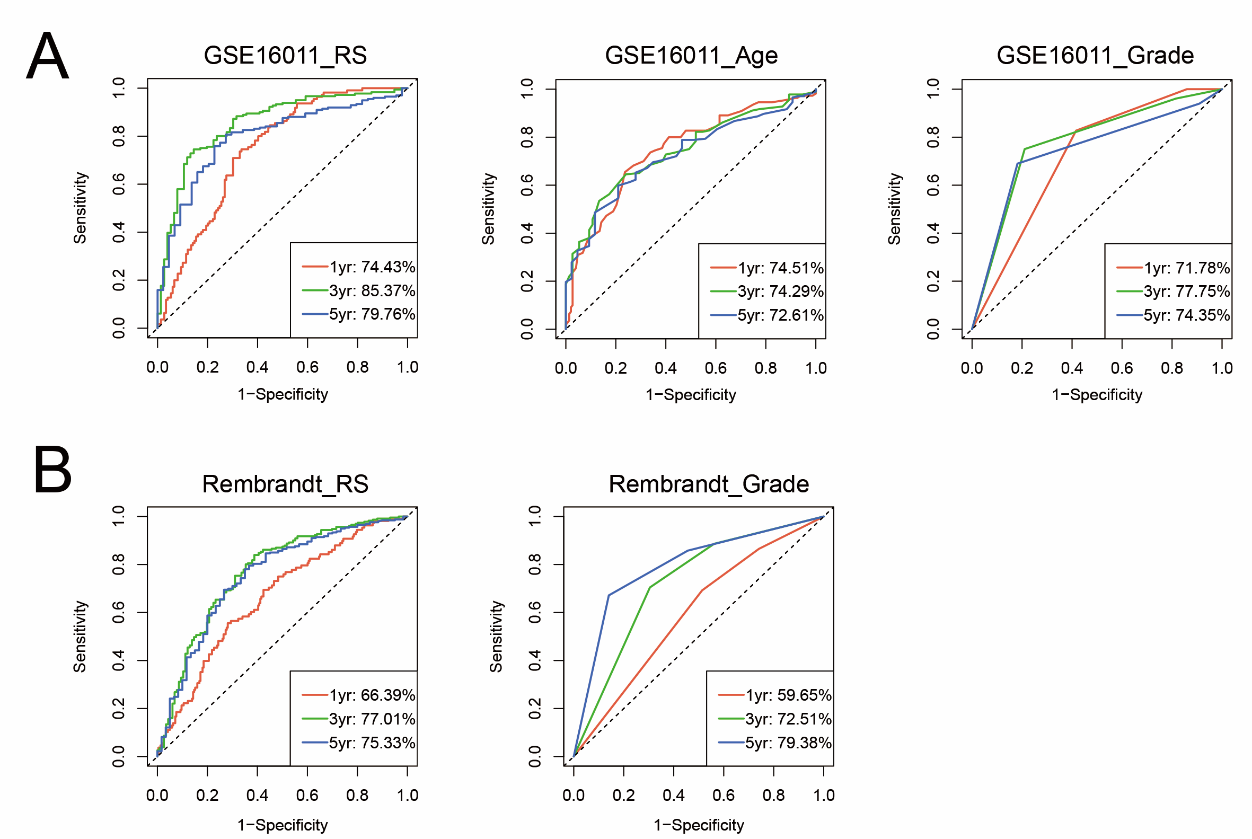


**Figure S5.** Corrplots show correlation between risk score (RS) and six immune cell types. Red indicates a positive correlation and blue indicates a negative correlation. The size of the circle and the intensity of the color are directly proportional to the correlation coefficient. The values represent the Pearson correlation coefficients.


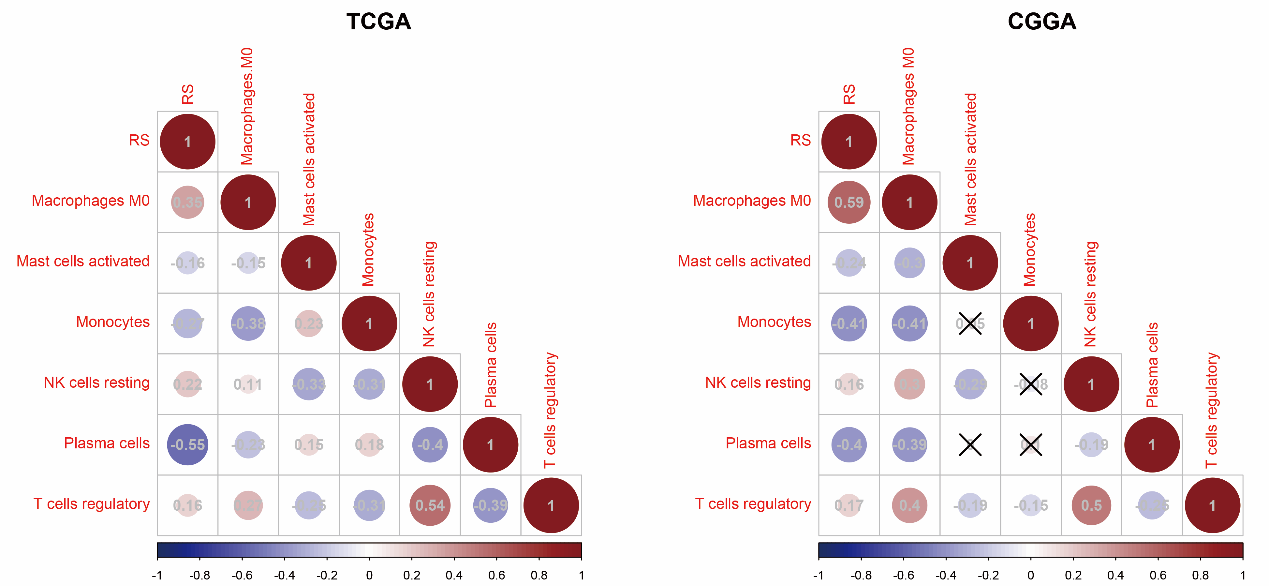


**Figure S6.** Corrplots show correlation between risk score (RS) and clinical molecular features. Red indicates a positive correlation and blue indicates a negative correlation. The size of the circle and the intensity of the color are directly proportional to the correlation coefficient. The values represent the Spearman correlation coefficients.

**
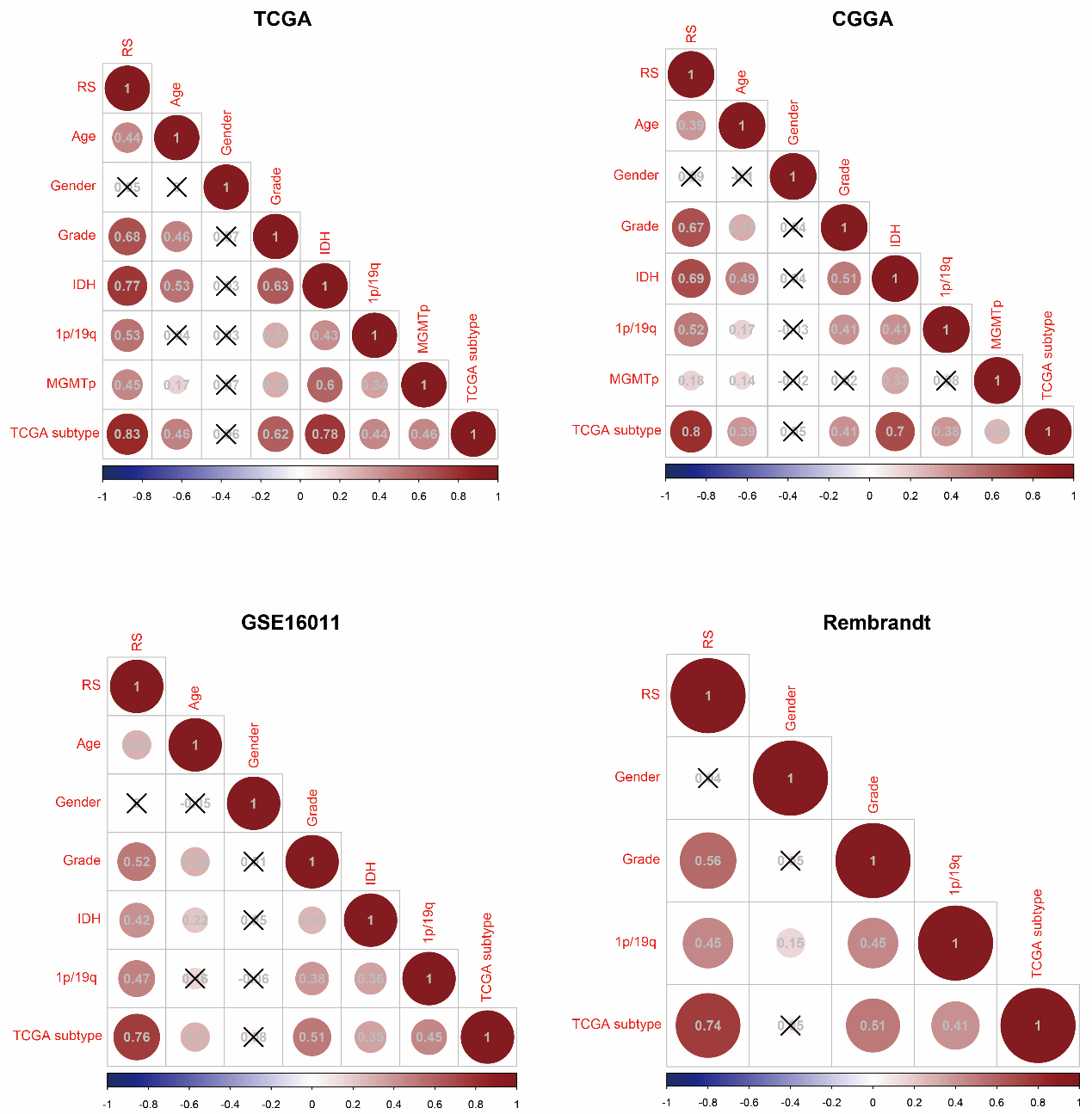
**

| **Table S1 Correlation between RS and clinicopathological factors of glioma patients** | | | | | | |
| --- | --- | --- | --- | --- | --- | --- |
|  | **GSE16011 dataset (n=268)** | | | **Rembrandt dataset (n=454)** | | |
| **Characteristics** | Low-risk group (n=134) | High-risk group (n=134) | P-Value | Low-risk group (n=227) | High-risk group (n=227) | P-Value |
| **Age** |  |  |  |  |  |  |
| Mean(range) | 48 (23-81) | 54 (14-81) | <0.001 | NA | NA |  |
| **Gender** |  |  |  |  |  |  |
| Female | 48 | 40 | n.s | 70 | 56 | n.s |
| Male | 86 | 94 |  | 112 | 110 |  |
| NA | 0 | 0 |  | 45 | 61 |  |
| **Grade** |  |  |  |  |  |  |
| 2 | 22 | 2 | <0.001 | 79 | 20 | <0.001 |
| 3 | 63 | 22 |  | 53 | 31 |  |
| 4 | 49 | 110 |  | 59 | 169 |  |
| NA | 0 | 0 |  | 36 | 7 |  |
| **IDH status** |  |  |  |  |  |  |
| Mutant | 56 | 24 | <0.001 | NA | NA |  |
| Wildtype | 51 | 85 |  | NA | NA |  |
| NA | 27 | 25 |  |  |  |  |
| **1p/19q status** |  |  |  |  |  |  |
| Codel | 41 | 5 | <0.001 | 30 | 3 | <0.001 |
| Non-codel | 41 | 47 |  | 76 | 116 |  |
| NA | 52 | 82 |  | 121 | 108 |  |
| **MGMT promoter** |  |  |  |  |  |  |
| Methylated | NA | NA |  | NA | NA |  |
| Unmethylated | NA | NA |  | NA | NA |  |
| NA |  |  |  | NA | NA |  |
| **TCGA subtype** |  |  |  |  |  |  |
| Proneural | 87 | 8 | <0.001 | 142 | 20 | <0.001 |
| Classical | 14 | 44 |  | 13 | 77 |  |
| Mesenchymal | 13 | 76 |  | 15 | 125 |  |
| NA | 20 | 6 |  | 56 | 5 |  |
